# Making the use of psychotropic drugs more rational through the development of GRADE recommendations in specialist mental healthcare

**DOI:** 10.1186/1752-4458-7-14

**Published:** 2013-05-02

**Authors:** Giovanni Ostuzzi, Irene Bighelli, Barbara-Vanessa Carrara, Nicola Dusi, Giuseppe Imperadore, Camilla Lintas, Francesco Nifosì, Michela Nosè, Carlo Piazza, Marianna Purgato, Raffaella Rizzo, Corrado Barbui

**Affiliations:** 1 WHO Collaborating Centre for Research and Training in Mental Health and Service Evaluation, Department of Medicine and Public Health, Section of Psychiatry, University of Verona, Verona, Italy; 2 Unit of Psychiatry II, Verona, ULSS20, Italy; 3 Unit of Psychiatry I, Verona, ULSS20, Italy; 4 Unit of Psychiatry IV, Verona, ULSS20, Italy

**Keywords:** Treatment guidelines, Knowledge transfer, Mental healthcare, Psychotropic drugs

## Abstract

**Introduction:**

In recent years the *Grading of Recommendations Assessment*, *Development and Evaluation* (GRADE) methodology has often been used by international or national health authorities, or scientific societies, for developing evidence-based treatment recommendations. However, the GRADE approach has never been used by practicing physicians who aim at harmonizing their prescribing behaviours paying due attention to the best available evidence. This paper describes the experience of a working group of psychiatrists who adopted the GRADE approach to develop clinical recommendations on the use of psychotropic drugs in specialist mental healthcare.

**Case description:**

The project was conducted in the Department of Mental Health of Verona, Italy, a city located in the north of Italy. At the beginning of 2012, psychiatrists with a specific interest in the rational use of psychotropic drugs were identified and appointed as members of a Guideline Development Group (GDG). The first task of the GDG was the identification of controversial areas in the use of psychotropic drugs, the definition of scoping questions, and the identification of outcomes of interest. The GDG was supported by a scientific secretariat, who searched the evidence, identified one or more systematic reviews matching the scoping questions, and drafted GRADE tables.

**Discussion and evaluation:**

On the basis of efficacy, acceptability, tolerability and safety data, considering the risk of bias and confidence in estimates, and taking also into consideration preferences, values and practical aspects in favour and against the intervention under scrutiny, a draft recommendation with its strength was formulated and agreed by GDG members. Recommendations were submitted for consideration to all specialists of the Department, discussed in two plenary sessions open to the whole staff, and finally approved at the end of 2012.

**Conclusion:**

The present project of guideline development raised several challenging and innovating aspects, including a “bottom-up” approach, as it was motivated by reasons that found agreement among specialists, those who developed the recommendations were those who were supposed to follow them, and values, preferences and feasibility issues were considered paying due attention to local context variables.

## Background

Clinical practice guidelines have progressively become a tool for supporting an evidence-based approach in health care. Guidelines are mostly seen as tools for making health practice more consistent and efficient and for narrowing the gap between what clinicians do and what scientific evidence supports [1-3].

In recent years new methodological approaches for aggregating, synthesising and grading the quality of evidence have progressively been developed, in order to support a transparent and methodologically sound production of clinical practice guidelines. One of these tools is the GRADE methodology (Grading of Recommendations Assessment, Development and Evaluation) [4-6]. This methodology has already been used to produce guidelines for several fields of medicine, including mental health care [7]. For example, WHO developed a model intervention guide within the mental health Gap Action Programme (mhGAP) [8], which provides recommendations to facilitate care at first and second level facilities by the non-specialist health care providers in low and middle income countries.

However, the GRADE approach has never been used by practicing physicians who aim at harmonizing their prescribing behaviours paying due attention to the best available evidence. This paper describes the experience of a working group of psychiatrists who adopted the GRADE approach to develop clinical recommendations on the use of psychotropic drugs in specialist mental healthcare. Here we highlight how the GRADE approach was adapted to our local needs, and we raise for consideration some challenging features of the whole process.

## Case description

### Setting

This project was conducted in the area of Verona, a city located in the north of Italy (450,000 inhabitants). In this area the main agency providing psychiatric care for the adult population is the Department of Mental Health (DMH) [9]. The DMH is a unitary service, in which great emphasis is given to communication between all staff members and to integration between the various clinical activities. It comprises four inpatient units located in three general hospitals and a network of outpatient and community facilities. The inpatient units are open wards of 16 beds each and patients can be admitted on a voluntary or compulsory basis. The Section of Psychiatry of the University of Verona is actively involved in the DMH activities in terms of clinical, teaching and research activities. In the last ten years it developed skills in the production of clinical trials, Cochrane reviews, and evidence-based recommendations.

At the beginning of 2012 the DMH started a project aimed at producing evidence-based recommendations for the pharmacological treatment of complex clinical situations. Final goals were the implementation of a robust and shared methodology for choosing and managing pharmacological treatments, and the assessment of the impact of recommendations on prescribing behaviours.

### Working group composition

**Figure 1:**
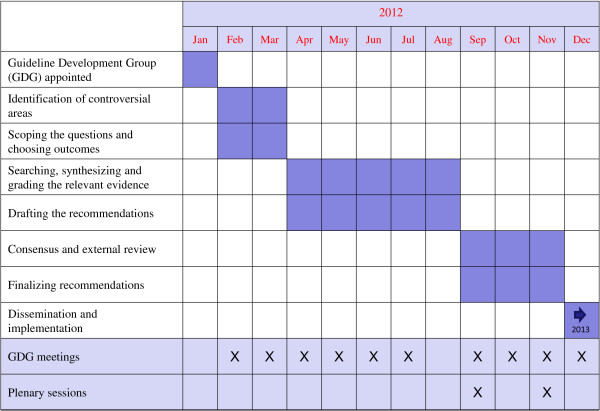
Diagram describing the process of recommendation development.

As initial step, psychiatrists with a specific interest in the rational use of psychotropic drugs were identified and appointed as members of a Guideline Development Group (GDG) (Figure 1). The group included at least one representatives from each of the four Mental Health Services of the DMH. The GDG was supported by a scientific secretariat, which included staff belonging to the Unit of Clinical Psychopharmacology and Drug Epidemiology of the University of Verona. The scientific secretariat was asked to provide scientific coordination and methodological support in the use of the GRADE approach. All GDG members and researchers of the scientific secretariat completed a form reporting potential financial and non-financial conflicts of interest.

### Formulating questions and choosing outcomes

The first task of the GDG was the identification of controversial areas in the use of psychotropic drugs. A clinician’s perspective was chosen, and a list of controversial areas was initially developed by the GDG and subsequently circulated by email to all specialists of the Department, with a request of providing feedback by highlighting the three topics perceived as the most relevant and where guidance was needed. Specialists were also allowed to make suggestions by adding other topics that were not part of the initial list. On the basis of this consultation process, the GDG selected the six most relevant topics, as follows:

1. Use of antipsychotics and benzodiazepines in patients with agitation and/or aggressive behaviour

2. Use of antipsychotics, antidepressants, benzodiazepines and mood stabilizers in patients with borderline personality disorder

3. Use of medicines in patients with unipolar depression:

3a. treatment strategies in patients who do not benefit from initial treatment

3a.  treatment strategies in patients with resistant depression

3a. duration of antidepressant treatment

4. Use of antipsychotics and antidepressants in elderly patients with:

4a. behavioural abnormalities

4a. depressive symptoms

5. Use of medicines during pregnancy

5a. antidepressants

5a. antipsychotics

5a. benzodiazepines

5a. mood stabilizers

6. Use of medicines in patients with medical comorbidities:

6a. cardiovascular problems

6a. Parkinson’s disease

6a. epilepsy

Each topic was reformulated using the PICO framework (Population, Intervention, Comparator, Outcome) into one or more scoping questions, in order to facilitate the process of searching and synthesizing the evidence. Finally, for each scoping question, the GDG discussed a number of possible outcomes of interest and, on the basis of relevance and clinical judgment, retained only those that were considered important or critical. These outcomes helped guide the subsequent phases of evidence retrieval, synthesis, and production of GRADE tables.

### Searching, appraising, synthesizing and grading the evidence

For each scoping question a member of the scientific secretariat was appointed as focal point. Focal points searched the evidence and identified one or more systematic reviews answering the scoping questions. We did not include primary studies. Existing guidelines based on newly performed systematic reviews, such as for example NICE guidelines, were included. Medline, Embase, Psychinfo, CINAHL the Cochrane Library and BMJ Clinical Evidence were routinely scanned to help identify pertinent systematic reviews. For efficacy, only systematic reviews of randomized trials were considered; for tolerability and safety outcomes, systematic reviews of observational studies were considered and, in selected cases, individual observational studies were included. Pragmatically, only recent reviews (three years) were selected, and we gave priority to Cochrane reviews, assuming a higher methodological standard and considering that the standard of reporting the results of Cochrane reviews is particularly suitable for producing GRADE tables. We matched for each outcome one systematic review. If more than one systematic review was available, sometimes providing conflicting results, this was recorded using the footnote tool of GRADE tables. Focal points critically appraised the evidence and drafted GRADE tables using the software GRADEpro [10]. As rating the quality of evidence for each included outcome is highly subjective, in order to increase consistency and reliability, the secretariat followed the instructions used by WHO in the mhGAP project [11].

**Figure 2:**
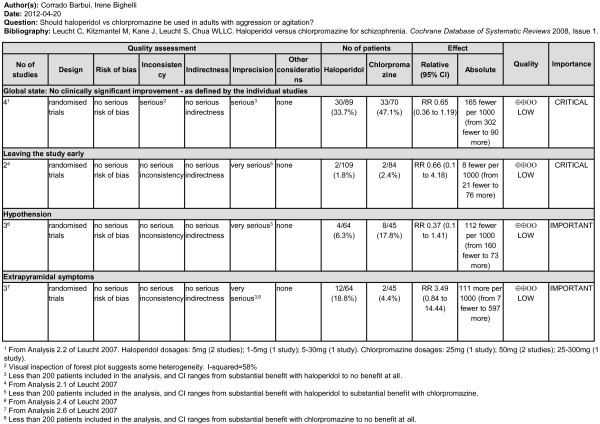
Example of GRADE table summarizing the evidence on haloperidol versus chlorpromazine in the treatment of patients with acute agitation.

For each scoping question a scoping document including a PICO table, a list of included systematic reviews, GRADE tables and additional evidence that was not graded, was drafted and analytically discussed by the GDG. If the production of GRADE tables was not feasible, a narrative approach was followed. Figure 2 provides an example of a GRADE table. It summarises the evidence on the beneficial and harmful effects of using haloperidol versus chlorpromazine in the treatment of patients with acute agitation. An interesting aspect is that the selected systematic review included different data for different outcomes: for example, for efficacy a total number of four studies were included, while for tolerability outcomes two or three studies contributed to the evidence base. As a consequence, the column “quality” reports a judgment on the confidence in the overall treatment estimate for each included outcome, as according to GRADE quality does not refer to the included systematic review, but rather to the confidence in estimate for each included outcome.

### Drafting the recommendations

The GDG critically appraised the evidence and suggested revisions to the GRADE tables and to the whole scoping documents. In addition, during GDG meetings, considerations on values and preferences, practical aspects such as drug labels, approved indications and safety warnings from regulatory agencies, feasibility issues and local aspects, were raised and extensively discussed. These considerations were incorporated into the scoping documents as key aspects to consider in addition to the background evidence when drafting a recommendation. Again, we followed the instructions used by WHO in the mhGAP project [11].

On the basis of efficacy, acceptability, tolerability and safety data, and considering the risk of bias and confidence in estimates, and taking also into consideration preferences, values and practical aspects in favour and against the intervention under scrutiny, a draft recommendation with its strength was formulated, agreed by GDG members, and reported at the end of each scoping document. Following WHO methodology, recommendation strength was categorised into standard or strong. Only when the GDG was very certain that benefits outweighed risks and burdens a recommendation was rated as strong.

### Consensus and external review

Recommendations were submitted for consideration to all specialists of the DMH and discussed in two plenary sessions open to the whole staff. On the basis of feedback and suggestions obtained during these plenary sessions, final recommendations were drafted by the GDG. In addition, remarks and clinical considerations that were considered useful to contextualise the recommendations were added, although these were kept separated from the formally approved recommendations. The revised versions were circulated to all specialists for consensus and final approval.

### Dissemination

From each scoping document we extracted the PICO table, a summary table of efficacy and tolerability data, a summary table of additional considerations on values, preferences and feasibility issues, and the recommendation with its remarks. These extracts were included in an official document that was disseminated to all staff both in print and electronic format. A professional designer was involved in its production in order to maximise readability and usefulness.

### Monitoring

The project started in January 2012, and in January 2013 we were able to disseminate the recommendations (Figure 1). In order to evaluate their impact on prescribing practices, a list of indicators have been identified which descriptively monitor the degree of coherence between what is reported in the guidelines and what is actually done in clinical practice [12,13]. Using pharmacy databases, a local Psychiatric Case Registry and other administrative sources of data, we aim to compare prescribing practices before and after the dissemination of guidelines.

## Discussion and evaluation

### Challenging aspects of the project

The present project of guideline development raised several challenging aspects. A first issue is the GDG composition. According to standard GRADE procedure for guideline development, a multi professional GDG with physicians, nurses, pharmacists, methodologists, patients, representatives of patient and family associations, hospital policy makers and other civil society representatives is suggested in order to keep into consideration different perspectives and input [14,15]. By contrast, only specialists working in the psychiatric facilities of the local catchment area were selected. This is indubitably a study limitation, which was carefully considered before the GDG was appointed. However, we reasoned that the present project was aimed at providing a concrete answer to a specific need expressed by specialists, that is to harmonize prescribing practices and give suggestions for controversial clinical issues. Given such a clear mission, strongly focused on specialists as target audience, it came naturally to involve them all in the production of recommendations that would regulate their own behaviour. Researchers with a long experience in the field of clinical trials, systematic reviews and guideline production were nevertheless actively involved, but only as members of the scientific secretariat.

A second challenge was that, according to GRADE methodology, recommendations should be formulated keeping into consideration not only the evidence base, but also values, preferences and feasibility issues [16]. This aspect was given much value in the present project, and long discussions concerned regulatory issues, warnings issued by European or national or regional authorities and on the pros and cons of off-label prescribing. Other discussions concerned value judgements, such as the relevance of treating a specific clinical condition despite the lack of background evidence, and feasibility considerations, often related to local context variables, such as for example medication availability in different psychiatric settings [17]. A challenge of this approach was the relative weight that these aspects should receive in comparison with aspects related to the evidence base. We note that in some circumstances there was a risk of generating recommendations only loosely connected with the background evidence. For example, while the evidence base suggests that second-generation antipsychotics may be beneficial in treatment-resistant depression with no reason to prefer one specific drug, the GDG pointed out that in Italy only quetiapine has a formal indication for use in this condition, and this was incorporated into the recommendation.

Other challenging aspects refer to the GRADE methodology [11]. Indirectness was a major issue, as the GDG realized that very often the patient population included in clinical trials did not match with the target population. Second, for several outcomes inserted in GRADE tables no evidence was available, as trials usually employ outcome measures that can easily be measured but may not always be considered that relevant in clinical practice. The extensive use of rating scales in psychiatry is a paradigmatic example of this, as these scales are seldom used in clinical practice and the clinical interpretation of differences in mean scores is rather obscure [18]. The GDG noted some exceptions, though. For example, while many trials conducted in patients with aggressive behaviour used as primary outcome a rating scale score, some studies with a very pragmatic design measured efficacy with a clinically sound indicator, which was “being asleep 15 minutes and 4 hours after the intervention” [19-21]. Third, for some scoping questions evidence was not available in the form of systematic reviews or randomized trials, for example when tolerability of psychotropic drugs in pregnant women or in patients with medical comorbidity were addressed. For these questions, data from observational studies and case-series were retrieved, but rarely these have been systematically reviewed, and GRADE tables could not be produced. As we had no resources to conduct new systematic reviews of observational studies, a narrative approach was employed in these situations, but we acknowledge it was far from being optimal as some studies might have been missed and quality of evidence was not explicitly assessed.

### Innovative aspects of the project

Clinical guidelines are usually developed by national or local health authorities, international or national scientific societies, non-governmental organizations [22]. This approach has been described as “top-down”. Although guideline development or endorsement by high reputation organizations, such as WHO for example, may theoretically increase their uptake in clinical practice [23], there are disadvantages with this approach. First, those who develop the guidelines are not those who are supposed to follow them, who are only asked to work to standards that were set by others; second, the production of guidelines is usually motivated by reasons that may not have found agreement among specialists, including financial control and medico-legal aspects; third, a “top-down approach” can hardly include values, preferences and feasibility issues that pay due attention to local variables.

By contrast, in the present project a “bottom-up” approach was followed, starting from an explicit request formulated by specialists who felt a need of implementing a more rational and careful use of psychotropic drugs. The whole project has been developed on these grounds, from the composition of the GDG to the selection of topics, from the involvement of specialists in the critical appraisal of the evidence base to the inclusion of local feasibility consideration. Specialists were involved both in the technical process (systematic reviews of relevant evidence) and in the social process (interpretation of the results of the systematic review) of guideline development. This approach provided doctors with an opportunity of training their ability to manage the evidence base, which is not straightforward for most doctors, and their attitude to explicitly draft recommendations on prescribing behaviours. The educational features of this project were formally acknowledged by local institutions which provided Continuing Medical Education (CME) credits to all participants.

We still do not know if this “bottom-up” approach will enhance guideline uptake in practice [24]. However, data have consistently shown that guidelines are still underused by practitioners [25,26], and effective strategies for dissemination and implementation are needed. This holds true in health care in general [27] and in mental healthcare [28]. The work described here attempted to engage specialists since the very initial steps of guideline production, as an effort to maximise implementability. Hopefully, the monitoring phase that is currently ongoing will clarify whether this process is likely to be an effective implementation strategy.

## Conclusions

The present project will be replicated in 2013, appointing different psychiatrists of the DMH as members of the GDG, and focusing on another set of controversial topics. The GDG will also regularly check the need of updating the existing guidelines. The scientific secretariat will be unchanged and will assure continuity in terms of methodology and overall process. Although we acknowledge the risk of guideline proliferation with an increasing issue of quality and potential conflicts of interest [29], we note that the experience described in the present paper suggests that physicians may effectively organize themselves becoming proactively involved in the production of clinical practice recommendations that adhere to internationally accepted quality standards, in the absence of financial or other kinds of conflicts of interest. We argue that the product of these initiatives should be offered to policy makers as standards of reference.

## Consent

Not applicable.

## Competing interests

The authors declare that they have no competing interests.

## Authors’ contributions

GO drafted the manuscript, IB co-led the drafting of the manuscript, CB provided input and edits and critically revised the manuscript. All authors participated in the project, and were members of the Guideline Development Group. All authors read and approved the final manuscript.
